# Improving Aviation Safety through Modeling Accident Risk Assessment of Runway

**DOI:** 10.3390/ijerph17176085

**Published:** 2020-08-21

**Authors:** Yaser Yousefi, Nader Karballaeezadeh, Dariush Moazami, Amirhossein Sanaei Zahed, Danial Mohammadzadeh S., Amir Mosavi

**Affiliations:** 1 School of Civil Engineering, Iran University of Science and Technology, Tehran P.O. Box 16765-163, Iran; yaser.yousefi@ymail.com; 2 Faculty of Civil Engineering, Shahrood University of Technology, Shahrood P.O. Box 3619995161, Iran; n.karballaeezadeh@shahroodut.ac.ir; 3 Department of Elite Relations with Industries, Khorasan Construction Engineering Organization, Mashhad P.O. Box 9185816744, Iran; ah.sanaei@toos.ac.ir (A.S.Z.); danial.mohammadzadehshadmehri@mail.um.ac.ir (D.M.S.); 4 Department of Civil Engineering, Faculty of Montazeri, Khorasan Razavi Branch, Technical and Vocational University (TVU), Mashhad P.O. Box 9176994594, Iran; dariush-moazami@tvu.ac.ir; 5 Toos Institute of Higher Education, Khorasan Razavi, Mashhad P.O. Box 9188911111, Iran; 6 Department of Civil Engineering, Ferdowsi University of Mashhad, Mashhad P.O. Box 9177948974, Iran; 7 Department of Civil Engineering, Mashhad Branch, Islamic Azad University, Mashhad P.O. Box 9187147578, Iran; 8 Faculty of Civil Engineering, Technische Universität Dresden, 01069 Dresden, Germany; 9 Institute of Research and Development, Duy Tan University, Da Nang 550000, Vietnam; 10 Department of Informatics, J. Selye University, 94501 Komarno, Slovakia

**Keywords:** aviation safety, airport safety, runway, accident risk assessment, airport design, runway excursion, accident research, digital health, public health, mobility, transportation, airplane collision, accident injuries prevention, accident modeling, air traffic

## Abstract

The exponential increase in aviation activity and air traffic in recent decades has raised several public health issues. One of the critical public health concerns is runway safety and the increasing demand for airports without accidents. In addition to threatening human lives, runway accidents are often associated with severe environmental and pollution consequences. In this study, a three-step approach is used for runway risk assessment considering probability, location, and consequences of accidents through advanced statistical methods. This study proposes novel models for the implementation of these three steps in Iran. Data on runway excursion accidents were collected from several countries with similar air accident rates. The proposed models empower engineers to advance an accurate assessment of the accident probability and safety assessment of airports. For in-service airports, it is possible to assess existing runways to remove obstacles close to runways if necessary. Also, the proposed models can be used for preliminary evaluations of developing existing airports and the construction of new runways.

## 1. Introduction

Progress of the aviation industry and airport services is considered essential for economic and social development [1]. Despite the numerous social and economic benefits, the expansion of air transport is confronted with several public health challenges [2]. The aviation accident is one of these problems. In addition to threatening human lives, accidents are often associated with severe environmental and pollution consequences [3]. Therefore, the environment and safety have always been of interest to engineers in this industry [1,2,3,4]. Process optimization can be effective in solving these problems and their impact on public health [2,5]. Accordingly, there is an increasing demand for the development of environmentally friendly and sustainable airports with minimal environmental impact [6]. On the other hand, the rapid transition of passengers and goods and developments of countries are closely linked to the efficiency of their transportation systems. For example, transportation activities allocate itself more than 9.1% of the Gross Domestic Product (GDP) and involve close to 3 million jobs in Iran. In various human societies, aviation is regarded as one of the key industries in terms of features such as security, speed, and the attractiveness of the airline industry tourism [7]. As the space of take-off and landing aircraft, the runway is one of the most important parts of an airport. Any accident at the runway will result in threaten public health, reducing airport functionality, and resulting in service disruption. Also, accidents can harm the environment in the surrounding of the runway [8,9]. The idea of getting air accident rates down to zero is very naive and unbelievable, but trying to prevent them will increase air transportation safety. Therefore, identifying obstacles, examining the location constraints, and determining the optimum location of the runway are among the essential requirements of each country’s aviation organization. It is expected that there will be perfectly safe conditions when landing and taking-off for each aircraft. Factors such as inclement weather, poor visibility, obstacles, lack of familiarity with the airport, confusion, fatigue, and disruption to Air Traffic Control (ATC) may cause an accident at the airport [10,11]. In general, runway accidents are divided into two main categories [8,12,13,14]:

• Runway Incursion (RI)

These accidents occur due to the improper positioning of a plane, vehicle, or human in a protected area for landing and take-off [15,16]. In other words, RI is an accident that occurs when a plane crashes into another plane or vehicle, or human at the runway. The three main causes of RI accidents are non-compliance with ATC instructions, lack of familiarity with the airport, and non-compliance with standard operating procedures [17,18].

• Runway Excursion (RE)

RE means leaving the aircraft from the runway. This type of accident is likely to occur during both the take-off and the landing [19]. According to the International Air Transport Association Safety Report, REs caused 22% of all accidents over the 2010–2014 period [20]. According to aviation authorities, about 80 percent of RE accidents occur in landing operations [3]. Several factors contribute to a RE accident, the most important of which are weather, pilot, airport, and aircraft [19,21]. Depending on the exit area of the runway, RE accidents are divided into two groups [22,23]: Veer-Off (VO) and Over-Run (OR). In this paper, two types of RE accidents have been considered by the authors: Landing Overrun (LDOR) and Landing Veer-off (LDVO).

Because of the geographical extent of Iran and the underdevelopment of the rail and road transport systems, the appeal of using air transport has been increasing in this country during the last years. Currently, the Iran aviation industry suffers from poor service quality, long service life, lack of advanced navigation system, lack of proper planning, etc. Due to the exhaustion of the Iranian air fleet, the need to use risk assessment models at the airports of this country is very urgent. Therefore, the main purpose of this paper is to develop the final models for risk assessment in Iranian airports. The method of air accident risk assessment in scientific literature consists of three parts [22]: Modeling the probability of accidents, Modeling the location of accidents, and Modeling the consequences of accidents. For this purpose, modeling of probability, location, and consequences of accidents has been performed. Due to the limited number of data, all data were used in the model construction phase, and validation of models was performed with the help of statistical tests: Analysis of Variance (ANOVA) and Residual Diagnostic. For a practical examination of the models, two airports in Iran were evaluated by these models, Mehrabad in Tehran and Hasheminejad in Mashhad. By using these models, engineers can accurately analyze the existing runways and remove obstacles for reducing the number of accidents. Therefore, proposed models can help to increase safety and public health. Also, in new airports, the models help engineers to evaluate proposed scenarios to select the optimal location of runways. As a result, they can consider environmental aspects and choose the location where the future probably accidents will have less damage to environmental elements such as wildlife parks, old trees, historical monuments, etc. The rest of the paper is organized as follows: Section 2 presents the background of the study. Section 3 introduces the materials and methods of this study. This section includes all concepts related to accident modeling, how to collect data and introduce the airports under study. Then, we show the results and discuss them in Section 4. Finally, Section 5 presents our conclusion.

## 2. Literature Review

Risk evaluations are used in many parts of aviation. Little information is available for evaluating the risk of accidents at airports or near them. Previous relevant studies can be classified into four parts [22]: operational risk, facility risk, airport design, and third-party risk. The focus of this paper is on third-party risk. The most important and well-known examples of third-party risk are the Airport Cooperative Research Program (ACRP) reports (Reports 3 [22] and 50 [24]). A review of relevant studies is provided below. In 2001, the Norwegian Civil Aviation Authority evaluated the risk of veer-off. They found that the probability function formation for veer-off accidents is exponential (see Equation (1)) [25]:

(1) $$P\left(x\right)=e^{-{\mathrm{ax}}^{n}}$$

where P is the probability that the aircraft at the end of the veer-off is *x* meters away from the runway centreline, while a and n are coefficients that depend on the conditions at the examined contour. The distance x is calculated from the center of gravity of the aircraft. Moretti et al. calculated the coefficient constant of Equation (1) for two conditions, including take-off and landing (see Equations (2) and (3)) [26]:

(2) $$P_{\mathrm{landings}}=e^{-0.0219x}$$

(3) $$P_{\mathrm{take}-\mathrm{offs}}=e^{-0.0143x}$$

**Figure 1:**
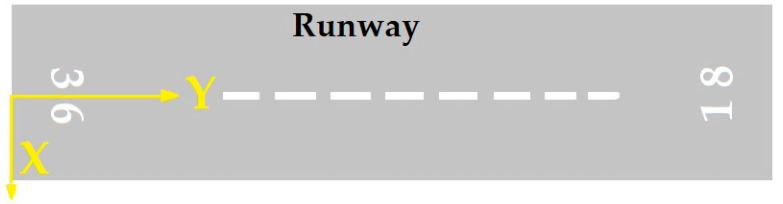
The defined Cartesian system for veer-off modeling in Table 1.

In another study, Moretti et al. selected an airport in Italy as a case study and calculated a veer-off risk assessment for 1500 points around that airport’s runway [27]. Their modeling results are presented in Table 1. In this table, P is the frequency of an aircraft running beyond a certain distance, x, measured from the runway centreline, according to the Cartesian system presented in Figure 1.

Kirkland et al. proposed a methodology for risk assessment of runway landing overruns. Their proposed methodology included three main models as described in [28]:

- Modeling the probability of a landing overrun
- Modeling the wreckage location
- Modeling the damage consequences of overruns

Table 2 shows the details of this methodology.

**Figure 2:**
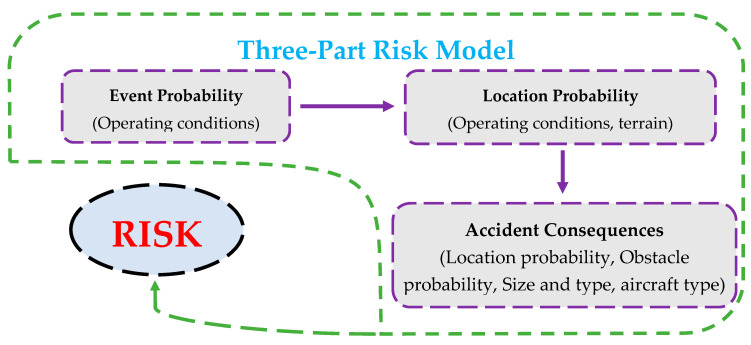
Risk modeling process in ACRP Report 3.

In ACRP Report 3, Federal Aviation Administration (FAA) develops models for risk assessment of the probability, location, and consequences of landing overruns (LDOR), landing undershoots (LDUS), and take-off overruns (TOOR) accidents [22]. For this report, the study period was from 1982 to 2006 and in addition to the US, data from similar countries (Canada, Australia, France, Britain, New Zealand, Singapore, Ireland, and Spain) were used. In this report, the three-part process shown in Figure 2 is used to model the risk of accidents. Figure 2 was adapted from ACRP Report 3 [22].

In this report, logistic regression and exponential function were used to model even probability and location probability, respectively. For the third part, the probability of severe consequences modeling was performed using frequency and location models [22]. Using the results of the ACRP Report 3, Wong et al. conducted a risk assessment for the Runway Safety Area (RSA) and focused on accident probability modeling. They investigated LDOR, LDUS, TOOR, and Crash after Takeoff (TOC) accidents at US airports. The study period was from 1982 to 2002 and a total of 440 accidents were selected, including 199 LDOR cases, 122 LDUS cases, 52 TOOR cases, and 67 TOC cases [29].

In 2011, the FAA published ACRP Report 50. This report presented an improved model of the probability of accidents for LDOR, LDVO, LDUS, TOOR, and take-off veer-offs (TOVO). The study used 1031 accidents and 383 incidents at airports of US and similar countries. Normal operation data (NOD) from US airports, operations that did not cause any accidents or incidents, were also used in this study [24]. In addition to adding the LDVO and TOVO accidents, the variables and their levels have been modified in ACRP Report 50.

Based on the ACRP Reports 3 and 50, Ayres et al. (2013) have focused on two issues [30]:

- Presenting an airport risk assessment model to assess air traffic-related hazards at the airport
- Managing the RSA as a risk reduction measure.

In their study, five types of runway accidents including LDOR, LDVO, LDUS, TOOR, and TOVO were analyzed and modeled. In terms of the status of accident distribution in the end and edges of the runway, they used the exponential form to model the accident location [30]. The modeling results performed by Ayres et al. are presented in Table 3.

Jeon et al. investigated the impact of rainfall on runway accidents. Due to not considering the effect of rainfall (rain and snow) on the RSA risk assessment in the existing studies, they attempted to develop a new location model to account for this effect [31]. The results of their research for the types of accidents are presented in Table 4.

The runway risk assessment studies have always faced a major challenge called the variety of conditions affecting risk analysis. Various variables, including air traffic, aircraft type, and quality, build quality of Infrastructures, budget, maintenance quality, weather conditions, etc., vary from region to region. Because of these changes, engineers are forced to model for each region with the specific conditions of that region. Due to the lack of a comprehensive and practical risk assessment system for runways at Iranian airports, the authors were encouraged to address this gap. Therefore, the main purpose of the present study is to evaluate the RSA risk concerning RE accidents at Iranian airports. In terms of health and environmental problems due to air accidents, risk analysis and determination of optimum location for constructing or developing runways can help reduce these problems. One of the most important and valid methods of runway risk assessment is the method presented in the United States, ACRP Report 50. After reviewing the collected data and comparing their dispersion pattern to the American method, the authors decided to base their modeling process on this method.

## 3. Materials and Methods

**Figure 3:**
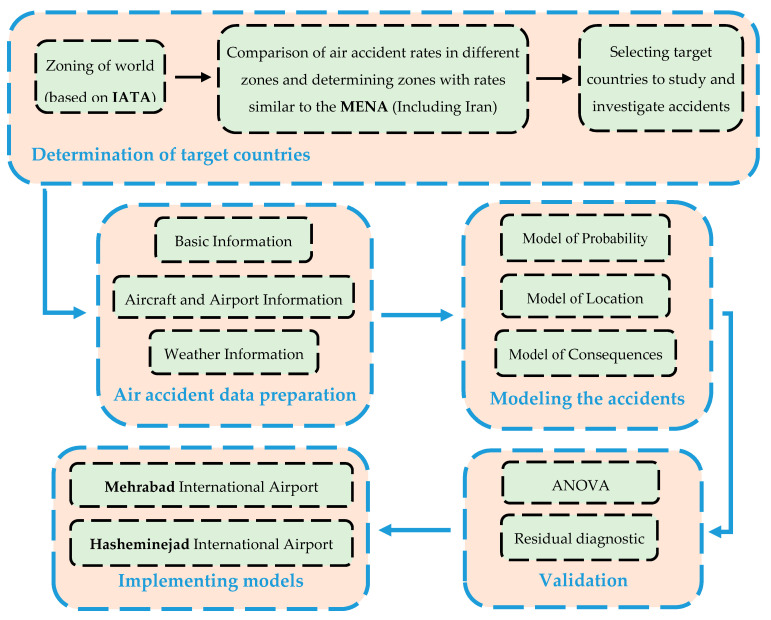
Research flow chart.

In this study, risk assessment modeling for LDOR and LDVO accidents is performed in three parts: Modeling the probability of accidents, Modeling the location of accidents, and Modeling the consequences of accidents. A comprehensive flowchart representing the framework of this research is presented in Figure 3. In the continuation of this section, the concepts related to runway risk assessment, data collection process, and specifications of the airports under study are presented in detail.

### 3.1. Risk Assessment of Runway Incidents

**Figure 4:**
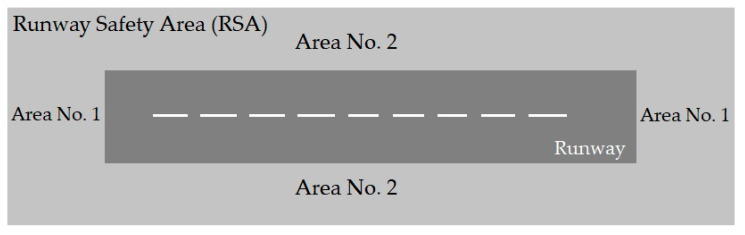
Runway Safety Area (RSA).

Runway Safety Area (RSA), Figure 4, is the sum of the area adjacent to the runway and the end area of the runway, which is built to reduce air accident damage [32,33,34,35,36]. The RSA standard dimensions have changed over the years and depend on the class of aircraft using the runway [24]. The standard dimensions have increased historically to serve faster and larger aircraft and to improved the safety of aviation users. The dimensions of RSA vary from 120 feet wide by 240 feet beyond the end of the runway to 500 feet wide by 1000 feet beyond the end of the runway [37]. In the 1960s, the FAA proposed an area that has 500 feet wide and extends 1000 feet beyond each end of the runway [24]. These dimensions are used as the standard in most runways. Based on Figure 4, areas (1) and (2) are provided to reduce OR and VO accident consequences, respectively.

#### 3.1.1. Accident Probability Modeling

Logistic regression is used to estimate the probability of accidents based on various factors, such as environmental factors. Logistic regression is similar to linear regression, except that in this type of prediction, the result for the level or levels of the dependent variable is two solutions [22]. The basic model used to create the accident probability model is as follows [22,24,31,38]:

(4) $$P\left\{\mathrm{Accident}\_\mathrm{Occurence}\right\}=\frac{1}{1+e^{-\left(b_{0}+b_{1}X_{1}+b_{2}X_{2}+b_{3}X_{3}+\ldots \right)}}$$

where P{Accident_Occurence} is the probability (0–100%) of an accident type occurring given certain operational conditions, X_i_ is independent variables (Table 5) including aircraft type, precipitation, crosswind, visibility, ceiling, etc., and b_i_ is regression coefficients. The values of b_i_ coefficients are given in Table 6. After obtaining the model coefficients (b_i_), by placing zero (base level) or one (other levels) numbers as an alternative to each of the independent variables (X_i_) in the model, it is possible to obtain the probability of different accidents. Table 5 and Table 6 were adapted from ACRP Report 50 [24].

#### 3.1.2. Accident Location Modeling

**Figure 5:**
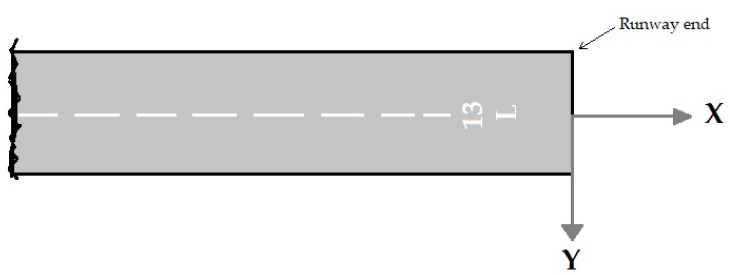
X-Y origin for LDOR accidents.

**Figure 6:**
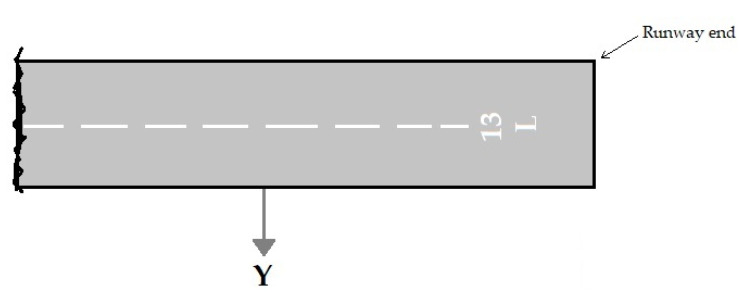
Y origin for LDVO accidents.

The likelihood of an accident in all areas around the airport is not equal. An accident near the runway is more likely to occur than the high distances from the runway. Therefore, the probability of accidents depends on the location of the accident. This dependence is expressed as a model of the accident location. The accident location model is based on past accident data [22,24]. According to the distribution of accidents towards the end and edges of the runway, the exponential function is used for modeling the accident location. The axis locations for measuring distances are viewed in Figure 5 and Figure 6. The nose wheel of aircraft is the reference location. These figures were adapted from ACRP Report 50 [24].

**Figure 7:**
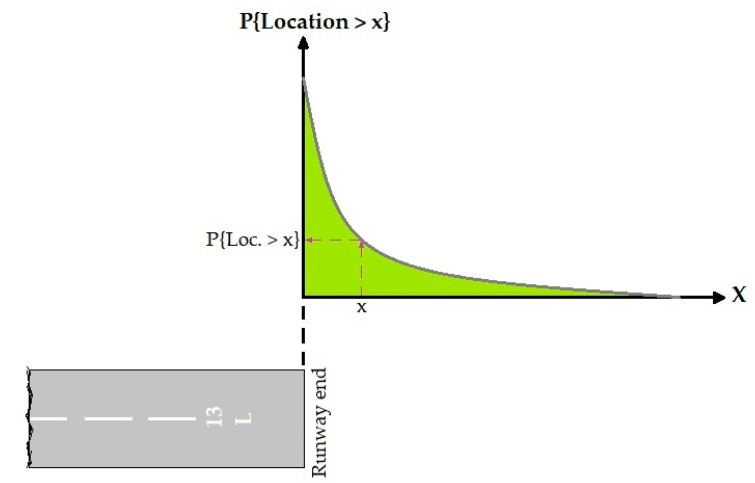
OR incidents model.

The basic model for the longitudinal distribution of OR accidents is [22,24]:

(5) $$P\left\{\mathrm{Location}>x\right\}=e^{-{\mathrm{ax}}^{n}}$$

where P{Location>x} is the probability the OR distance along the runway centerline beyond the runway end is greater than x and x a given distance beyond the runway end. a and n are regression coefficients. Figure 7 showed a typical longitudinal location distribution. This figure was adapted from ACRP Report 50 [24].

**Figure 8:**
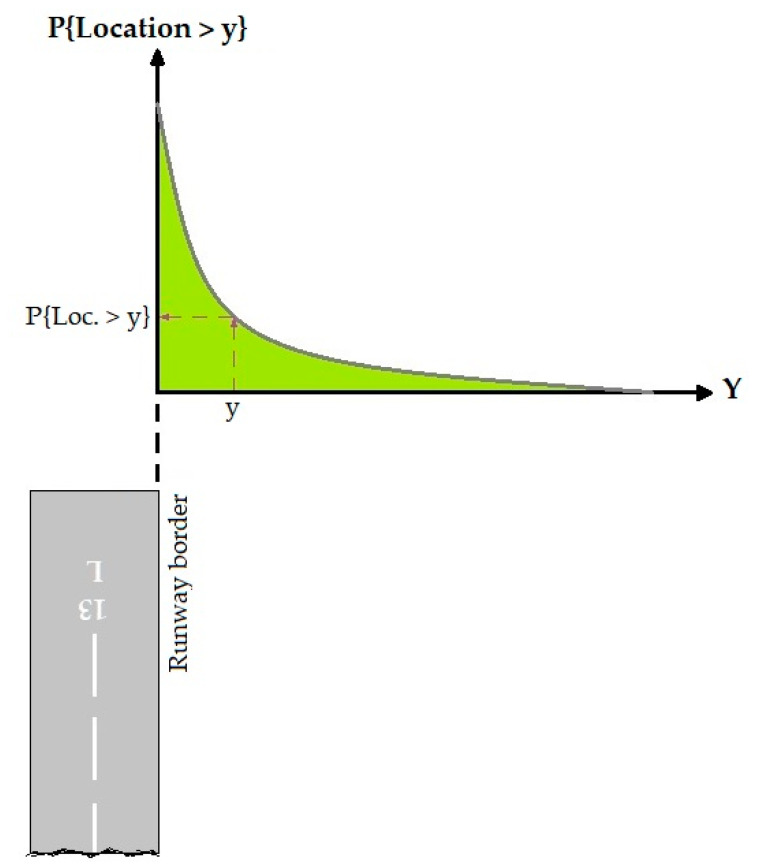
VO incidents model.

Equation (6) is used for transverse location distribution of OR and VO accidents:

(6) $$P\left\{\mathrm{Location}>y\right\}=e^{-{\mathrm{by}}^{m}}$$

where P{Location>y} is the probability that accident distance from the runway border (in VO accident) or centerline (in OR accident) is greater than y and y is a given distance from the extended runway centerline or runway border. b and m are regression coefficients. Figure 8 showed a typical transverse location distribution. This figure was adapted from ACRP Report 50 [24].

#### 3.1.3. Accident Consequences Modeling

The consequences of an accident are a function of several factors. In modeling the consequences of OR and VO accidents, the following variables are considered as effective factors in the accident severity [24]:

- Type, size, and location of the obstacle
- Aircraft speed and wingspan
- The number of obstacles and their location distribution

Based on the maximum speed of the aircraft, which has the least damage and casualties if it encounters an obstacle, obstacles are divided into four groups [24]:

Group one: Maximum speed is nill (such as cliff in RSA and concrete wall)

(1) Group two: Maximum speed is 5 knots (such as brick buildings)
(2) Group three: Maximum speed is 20 knots (such as ditches and fences)
(3) Group four: Maximum speed is 40 knots (such as frangible structure and approach lighting system)

**Figure 9:**
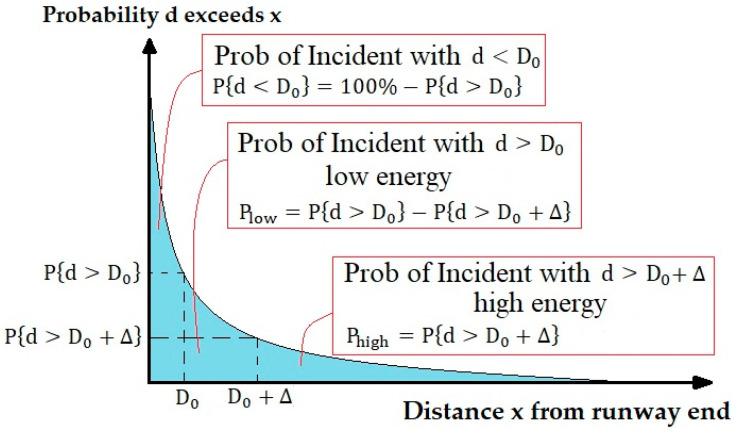
Modeling approach for OR accidents consequences.

The main idea in making the accident consequence model is to use models of accidents to estimate the accidents in which the aircraft has a lot of energy when it encounters an obstacle, thus producing severe consequences. This method was developed in ACRP Report 3 [22]. For a better understanding of this approach, note Figure 9.

In Figure 9, D_0_ is the distance to obstacle and d is the distance between the stopped aircraft and runway end. Δ value is predicted based on the rate of decrease in speed at different types of surfaces (paved, unpaved, and engineered materials arrestor system (EMAS)), the critical collision speed, and the size and type of obstacle [30].

Based on Figure 9, this modeling has three approaches [24]:

(1) The aircraft does not collide with the obstacle ($d<D_{o}$)
(2) The aircraft collides with the obstacle with low speed and energy ($d>D_{o}$)
(3) The aircraft collides with the obstacle with high speed and energy ($d>D_{o}+\Delta$)

**Figure 10:**
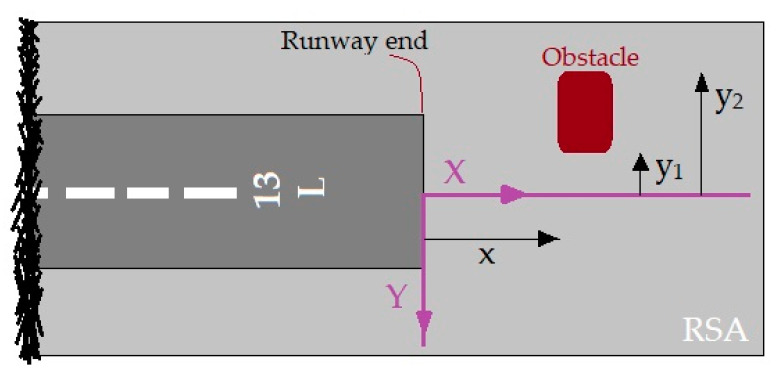
Modeling consequences.

**Figure 11:**
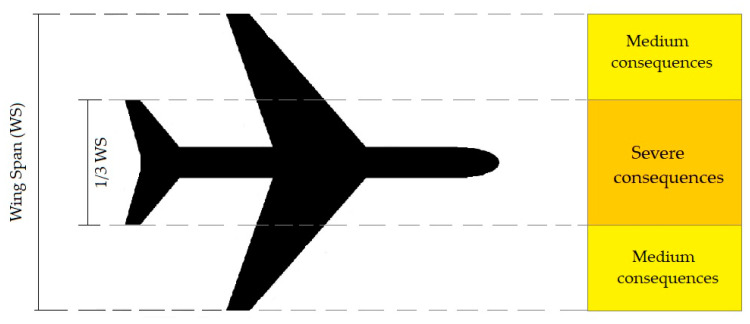
The impact of obstacle lateral location in accident consequences.

For completing the model, a link must be established between the longitudinal and transverse location distribution of accidents with the location, type, and dimensions of existing obstacles (See Figure 10 and Figure 11). These figures were adapted from ACRP Report 50 [24].

Based on Figure 10 and Figure 11, three general conditions can be expected [24,30]:

(1) Medium consequences when part of the obstacle is in the yellow area
(2) Severe consequences when part of the obstacle is in the orange area
(3) No consequences when the obstacle is out of the orange and yellow areas

**Figure 12:**
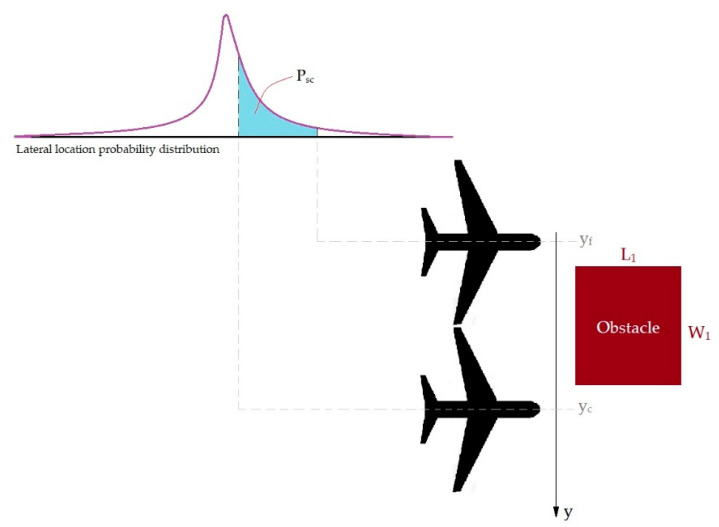
Modeling the possibility which an aircraft collides with an obstacle with severe consequences.

As can be seen in Figure 12, the W_1_ × L_1_ obstacle is located at a distance x and y of the runway threshold. It can be assumed that if the aircraft encounters an obstacle in the distance between y_c_ and y_f_, it will have severe consequences [24,30].

Based on the transverse distribution model of the accident location, the probability that the aircraft will be at the mentioned distance when exiting from the runway is [24]:

(7) $$P_{\mathrm{sc}}=\frac{e^{-{\mathrm{by}}_{c}^{m}}-e^{-{\mathrm{by}}_{f}^{m}}}{2}$$

where P_sc_ is the probability of high consequences, b and m are regression coefficients for transverse location model, y_c_ and y_f_ are critical location closest to the extended runway axis and farther from the extended runway axis, respectively.

By combining Equation (7), the longitudinal distribution model of accidents, and the possibility of multiple obstacles, the risk of accidents with severe consequences is determined by Equation (8) [24]:

(8) $$P_{\mathrm{sc}}=\overset{N}{\underset{i=1}{\sum }}\frac{\left(e^{-{\mathrm{by}}_{\mathrm{ci}}^{m}}-e^{-{\mathrm{by}}_{\mathrm{fi}}^{m}}\right)}{2}\times e^{-a{\left(x_{i}+\Delta_{i}\right)}^{n}}$$

where N is the number of existing obstacles, a and n are regression coefficients of longitudinal distribution model of accidents, and Δ_i_ is the location parameter for obstacle i.

### 3.2. Data Gathering

In this study, accident modeling is performed with the help of accident statistics in similar countries. According to the ACRP Report 3, the selection of similar countries has been done with the help of the standard of similarity of accident rates. Accordingly, the countries of the world are divided into eight separate regions [39]: Africa (AFI), Middle East and North Africa (MENA), Asia/Pacific (ASPAC), North Asia(NASIA), Commonwealth of Independent States (CIS), Europe (EUR), Latin America and the Caribbean (LATAM), and North America (NAM). Also, Table 7 shows the air accident rates per one million operations performed during 2013 and 2014, as well as the average for 2009–2013. Table 7 was extracted from the safety report 2014 [39].

Based on Table 7 and due to the nearness of air accident rates, MENA (including Iran), CIS, LATAM, and ASPAC zones have been selected as similar countries for RE accident data collection. A total of 98 countries have been selected in these four zones, of which 19 are in MENA, 12 in CIS, 33 in LATAM, and 34 in ASPAC. All RE accidents in the mentioned countries have been carefully examined and finally, the data of 57 countries have been selected. Table 8 shows the list of selected countries and the number of data extracted from each country. This table contains the data of LDOR, LDVO, TOOR, and TOVO accidents between 1990 and 2015.

**Figure 13:**
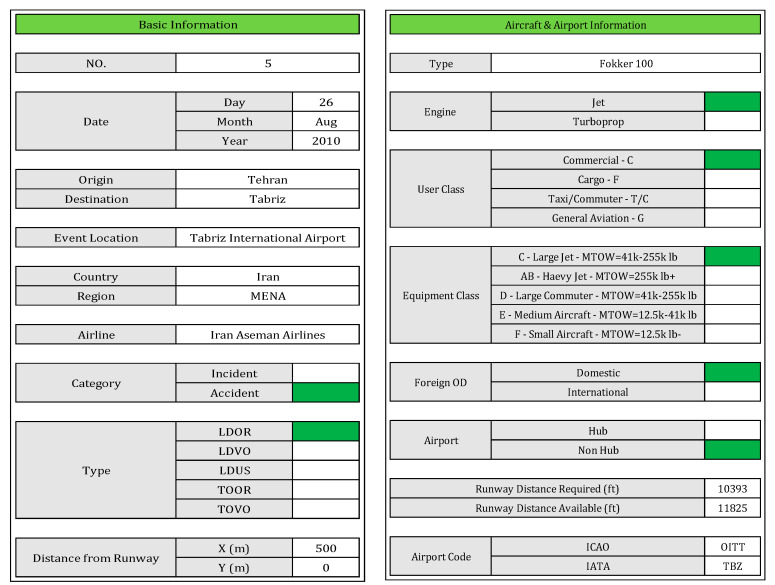

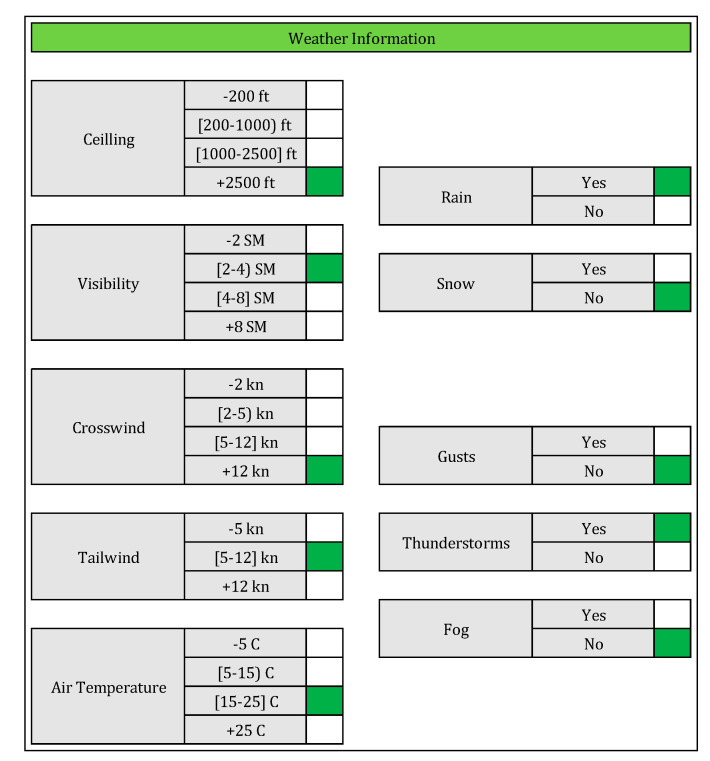
Initial forms for collecting and recording accidents, aircraft, airport, and weather conditions information.

Once the countries to collect data have been identified, three initial forms have been prepared including Basic Information, Aircraft and Airport Information, and Weather Information. Figure 13 shows these forms.

### 3.3. Airports Under Study

A study of the history of air accidents on Iranian airports shows that Mehrabad International Airport in Tehran and Hasheminejad International Airport in Mashhad have the largest share. Therefore, the accidents, the condition of the runways, and the obstacles around the runways of these two airports have been carefully examined.

#### 3.3.1. Mehrabad International Airport

**Figure 14:**
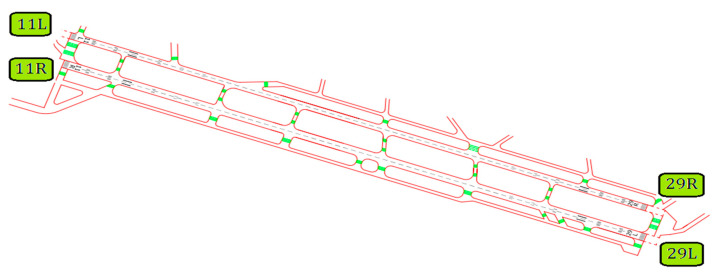
Mehrabad International Airport-Runways plan.

Mehrabad International Airport was built in 1938 in the western part of Tehran. A study of the presented statistics shows that in recent years, Tehran has been considered as the main, largest, and most important city in Iran, and some cases as the center of national and international exhibitions. The special conditions of Tehran have a direct impact on the performance and air traffic of the airport. Mehrabad Airport is currently the first airport in the country to receive about 12 million passengers. This airport has two parallel runways (see Figure 14).

Table 9 was extracted from the International Civil Aviation Organization (ICAO) [37] and shows the physical characteristics of runways in Mehrabad International Airport.

#### 3.3.2. Hasheminejad International Airport

**Figure 15:**
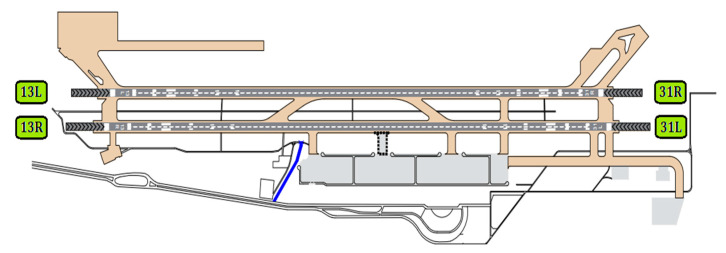
Hasheminejad International Airport-Runways plan.

Hasheminejad International Airport, in Mashhad, was built in 1951. This airport is the second busiest airport in the country after Mehrabad International Airport in Tehran. Hasheminejad International Airport has two parallel runways (See Figure 15). Also, Table 10 shows the physical characteristics of the runways. This table was extracted from ICAO [37].

## 4. Results and Discussion

**Figure 16:**
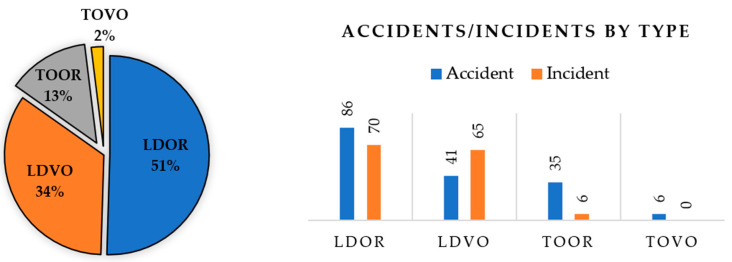
The events distribution (based on the type of RE accident).

The collected data are from 1990 to 2015. Undoubtedly, the number of RE accidents and incidents that occur during the period under review is much higher than the number of cases presented. However, the lack of registration of information about some of the accidents and incidents has made it impossible to use all of them in this research. A total of 309 events, including 168 accidents and 141 incidents, have been collected and classified. Figure 16 shows how these events are distributed.

As can be seen, the frequency of TOOR and TOVO data is low. Therefore, it is not possible to build a model based on this type of accident. As a result, further research is based on LDOR and LDVO accidents. Each event consists of 18 variables (16 variables related to the probability model and 2 variables related to the location model). Failure to record some variables has led to missing data in some collected cases. Finally, 128 and 143 data were used to construct the longitudinal and transverse LDOR accident model, respectively. Also, 49 data were used to construct the LDVO accident model. Since the probability model and the location model are made independently of each other, each of the selected events is at least appropriate for one of the models. Because of the limitations in the number of data, all of them were used in the construction of the model and the validation was done only by statistical tests.

### 4.1. Accident Probability Model

Due to the lack of a suitable database for NOD flights in Iran, it is not possible to build a model for the probability of RE accidents. Therefore, the studied airports will be analyzed based on the logistics regression model presented in the ACRP Report 50. Details of this model are provided in Section 3.1.1.

### 4.2. Accident Location Model

#### 4.2.1. Longitudinal Location Model for LDOR

**Figure 17:**
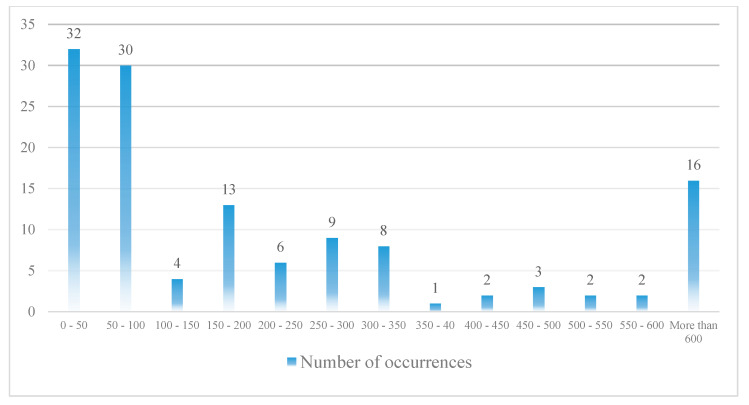
LDOR distribution in the X direction (Horizontal axis shows the distance (meter) from runway end).

**Figure 18:**
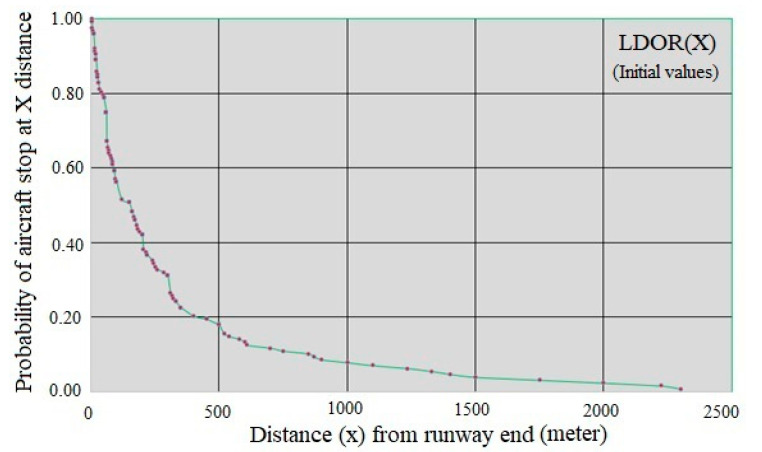
x curve versus P(X≥x) for LDOR.

In this section, the first location model for RE accidents is presented. The goal is to find F_X_(x) or the probability of x ∈ (a, b) for any desired value of a and b. According to the statistical preliminary concepts:

(9) $$F_{X}\left(x\right)=P\left(X\leq x\right)=1-P\left(X\geq x\right)$$

where F_X_(x) is the probability function x and P(X≤x) is probabilities of occurrence of X less than x. Figure 17 shows the location distribution of the studied LDOR accidents and incidents (indirect X) and Figure 18 shows the x curve versus P(X≥x).

Given that the relationship between the two variables is exponential, it is predicted that the P(X≥x) function can be estimated using exponential family regression. Based on Figure 18, the regression curve is considered as $P\left(X\geq x\right)=e^{-b_{0}x^{b_{1}}}+\varepsilon_{i}$, in which b_0_ and b_1_ are unknown parameters. Table 11 and Table 12 show the SPSS software output.

The R^2^ value of the model is equal to:

(10) $$R^{2}=1-\left(\frac{\mathrm{Residual}\;\mathrm{Sum}\;\mathrm{of}\;\mathrm{Squares}}{\mathrm{Corrected}\;\mathrm{Sum}\;\mathrm{of}\;\mathrm{Squares}}\right)=1-\frac{0.095}{10.876}=0.991$$

Based on the amount obtained for R^2^, it can be said that the adjusted regression can describe 99.1% of the sample information. Based on the calculations provided, the X-direction model of LDOR accidents and incidents is as follows:

(11) $$P\left(X\geq x\right)=e^{-0.012x^{0.804}}+\varepsilon_{i}$$

**Figure 19:**
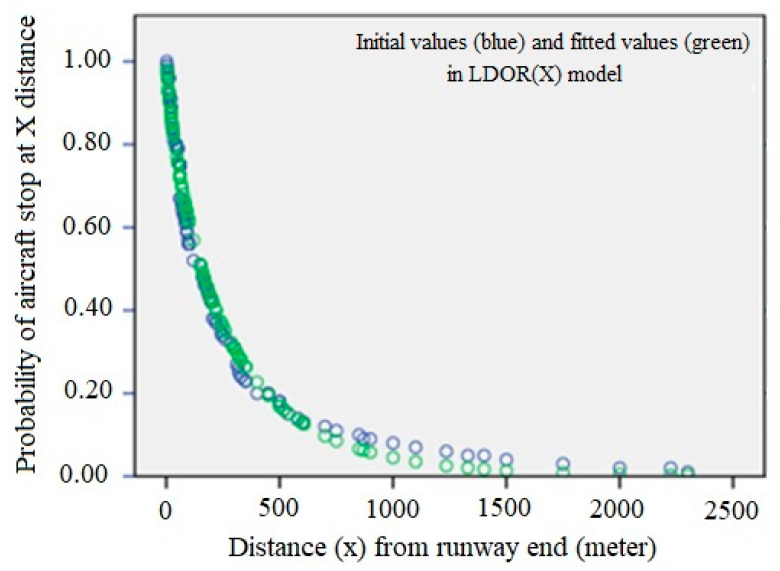
LDOR longitudinal regression curve in the location accident model.

Figure 19 shows the fitted curve for the LDOR model in the X-direction. In this figure, the green dots, the fitted values, and the blue dots are the initial values.

**Figure 20:**
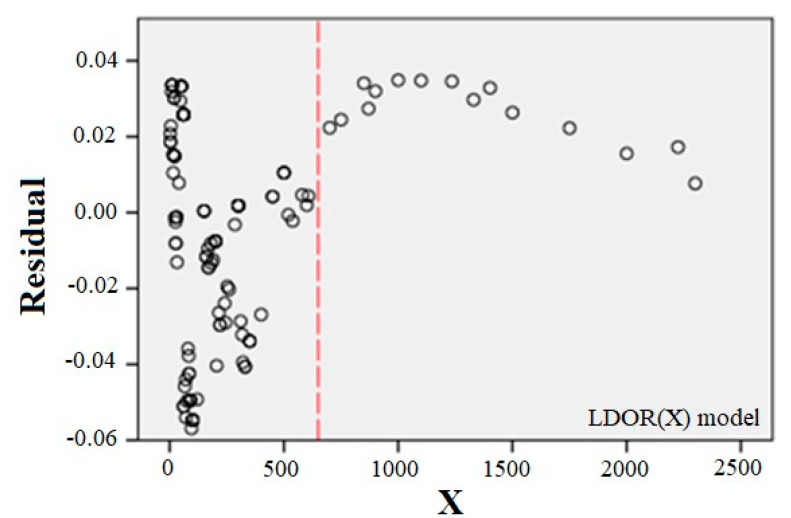
x scatter plot versus the residuals for the LDOR model in the X-direction.

At this step, two conditions are considered for the adequacy of the fitted model: the variance of the errors is constant and the errors are inconsistent. For this purpose, the scattering plot of the independent variable is drawn versus the residuals (Figure 20). If the model is correct, the residuals should not have a regular structure.

As can be seen, the model is shaky in the x > 650 range, which can be due to a lack of data in this range, but given that almost all data is less than 650, the adequacy of the model can be considered acceptable.

The probability of the plane overruns from the end of the runway, in the x-direction and during the landing, and locates in the range a to b from the end of the runway is as follows:

(12) $$F_{X}\left(x\right)=1-e^{-0.012x^{0.804}}$$

Consequently:

(13) $$P\left(a\leq x\leq b\right)=\int_{a}^{b}0.0097x^{-0.196}e^{-0.012x^{0.804}}\mathrm{dx}$$

#### 4.2.2. Transverse Location Model for LDOR

**Figure 21:**
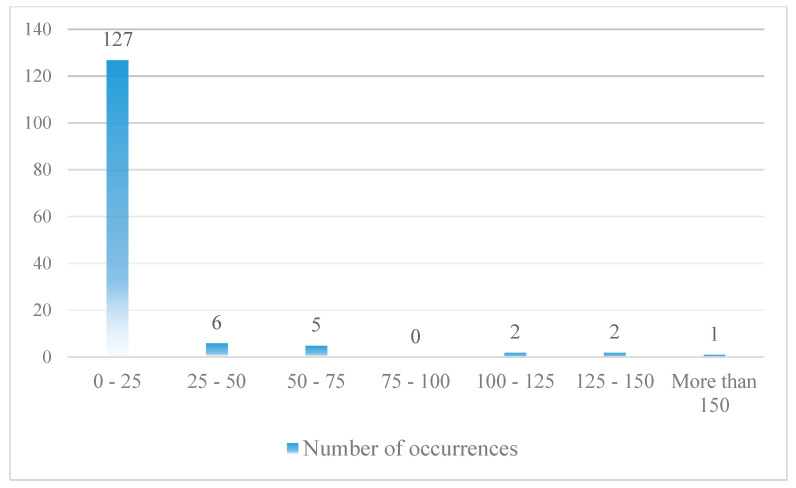
LDOR distribution in the Y direction (Horizontal axis shows the distance (meter) from runway centerline).

**Figure 22:**
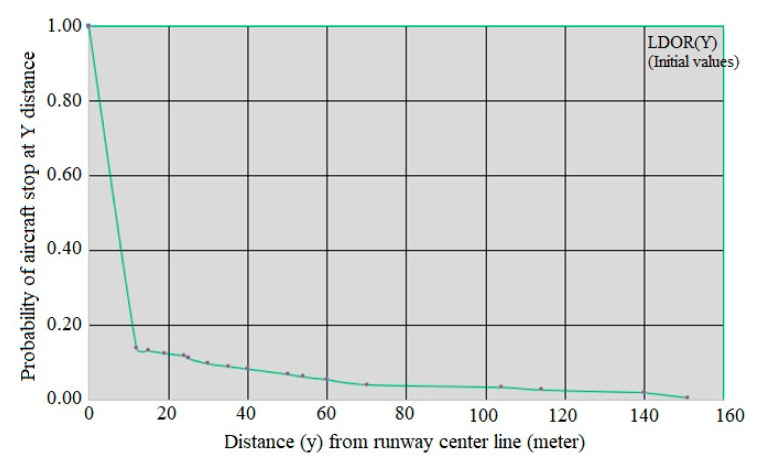
y curve versus P(Y≥y) for LDOR.

In this section, the second location model for RE accidents is presented. The goal is to find F_Y_(y) or the probability of y ∈ (a, b) for any desired value of a and b. According to the statistical preliminary concepts:

(14) $$F_{Y}\left(y\right)=P\left(Y\leq y\right)=1-P\left(Y\geq y\right)$$

where F_Y_(y) is the probability function y and P(Y≤y) is probabilities of occurrence of Y less than y. Figure 21 shows the location distribution of the studied LDOR accidents and incidents (indirect Y) and Figure 22 shows y curve versus P(Y≥y).

Like the model presented in the previous section, the relationship between the two variables is exponential, so it is predicted that the P(Y≥y) function can be estimated using family regression. Based on Figure 22, the regression curve is considered as $P\left(Y\geq y\right)=e^{-b_{0}y^{b_{1}}}+\varepsilon_{i}$, in which b_0_ and b_1_ are unknown parameters. Table 13 and Table 14 show the SPSS software output.

The R^2^ value of the model is equal to:

(15) $$R^{2}=1-\left(\frac{\mathrm{Residual}\;\mathrm{Sum}\;\mathrm{of}\;\mathrm{Squares}}{\mathrm{Corrected}\;\mathrm{Sum}\;\mathrm{of}\;\mathrm{Squares}}\right)=1-\frac{0.001}{14.767}=1.000$$

Based on the amount obtained for R^2^, it can be said that the adjusted regression can describe 100% of the sample information. Based on the calculations provided, the Y-direction model of LDOR accidents and incidents is as follows:

(16) $$P\left(Y\geq y\right)=e^{-0.932x^{0.275}}+\varepsilon_{i}$$

**Figure 23:**
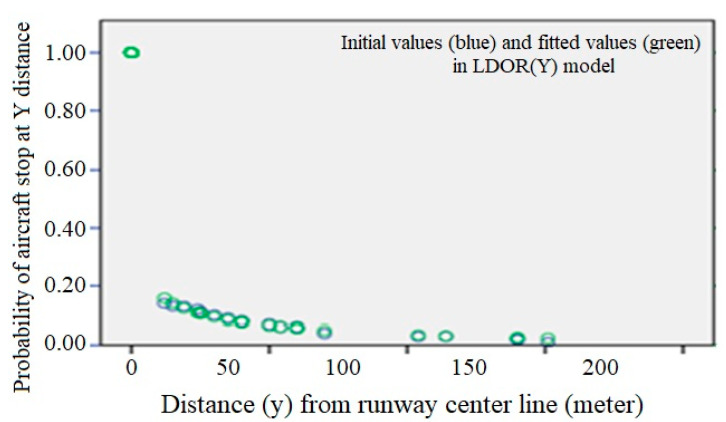
LDOR transverse regression curve in the location accident model.

Figure 23 shows the fitted curve for the LDOR model in the Y-direction. In this figure, the green dots, the fitted values, and the blue dots are the initial values.

**Figure 24:**
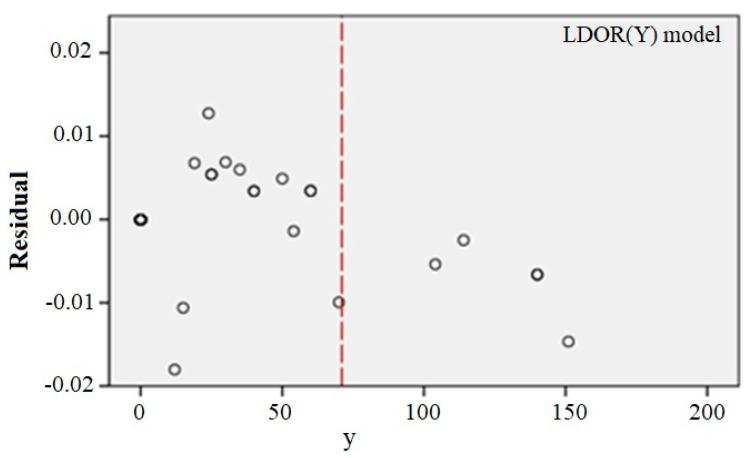
y scatter plot versus the residuals for the LDOR model in the Y direction.

At this step, two conditions are considered for the adequacy of the fitted model: the variance of the errors is constant and the errors are inconsistent. For this purpose, the scattering plot of the independent variable is drawn versus the residuals (Figure 24). If the model is correct, the residuals should not have a regular structure.

As can be seen, the model is shaky in the x > 72 range, which can be due to a lack of data in this range, but given that almost all data is less than 72, the adequacy of the model can be considered acceptable.

The probability of the plane overruns from the runway end, in the y-direction and during the landing, and locates in the range a to b from the runway centerline is as follows:

(17) $$F_{Y}\left(y\right)=1-e^{-0.932y^{0.275}}$$

Consequently:

(18) $$P\left(a\leq y\leq b\right)=\int_{a}^{b}0.2563y^{-0.725}e^{-0.932y^{0.275}}\mathrm{dy}$$

#### 4.2.3. Lateral Location Model for LDVO

**Figure 25:**
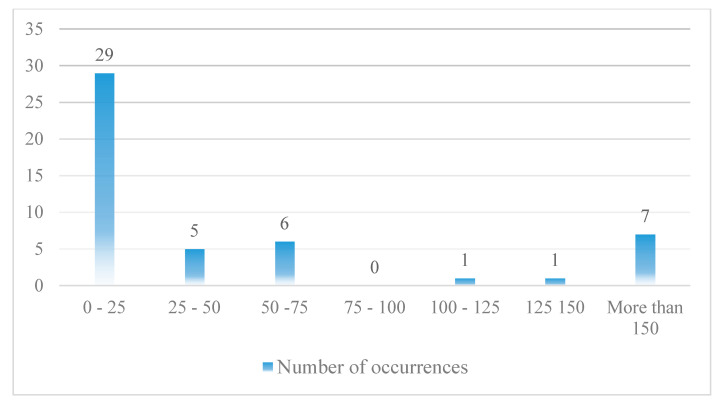
Location distribution of LDVO accidents (Horizontal axis shows the distance (meter) from runway edge).

**Figure 26:**
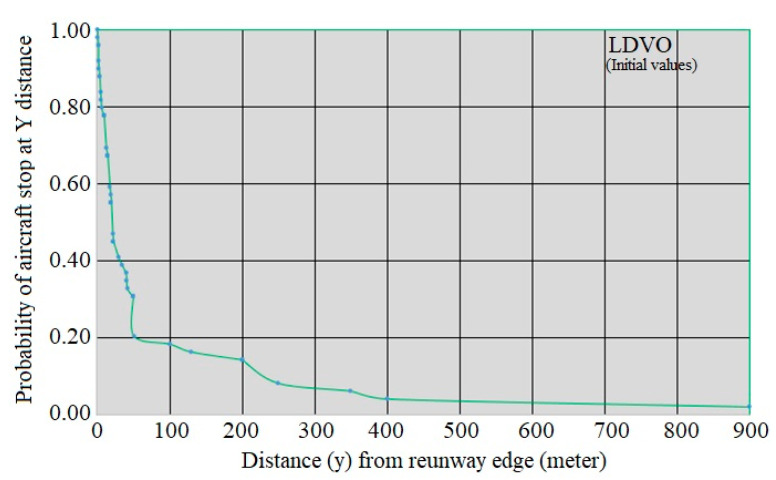
y curve versus P(Y≥y) for LDVO.

The third and final RE accident location model, the LDVO model, is presented in this section. The goal is to find F_Y_(y) or the probability of y ∈ (a, b) for any desired value of a and b. According to the statistical preliminary concepts:

(19) $$F_{Y}\left(y\right)=P\left(Y\leq y\right)=1-P\left(Y\geq y\right)$$

where F_Y_(y) is the probability function y and P(Y≤y) is probabilities of occurrence of Y less than y. Figure 25 shows the location distribution of the studied LDVO accidents and incidents (indirect Y) and Figure 26 shows y curve versus P(Y≥y).

Like the previous models, the relationship between the two variables is exponential, so it is predicted that the P(Y≥y) function can be estimated using family regression. Based on Figure 26, the regression curve is considered as $P\left(Y\geq y\right)=e^{-b_{0}y^{b_{1}}}+\varepsilon_{i}$, in which b_0_ and b_1_ are unknown parameters. Table 15 and Table 16 show the SPSS software output.

The R^2^ value of the model is equal to:

(20) $$R^{2}=1-\left(\frac{\mathrm{Residual}\;\mathrm{Sum}\;\mathrm{of}\;\mathrm{Squares}}{\mathrm{Corrected}\;\mathrm{Sum}\;\mathrm{of}\;\mathrm{Squares}}\right)=1-\frac{0.118}{4.078}=0.971$$

Based on the amount obtained for R^2^, it can be said that the adjusted regression can describe 97.1% of the sample information. Based on the calculations provided, the model of LDVO accidents and incidents is as follows:

(21) $$P\left(Y\geq y\right)=e^{-0.048y^{0.821}}+\varepsilon_{i}$$

**Figure 27:**
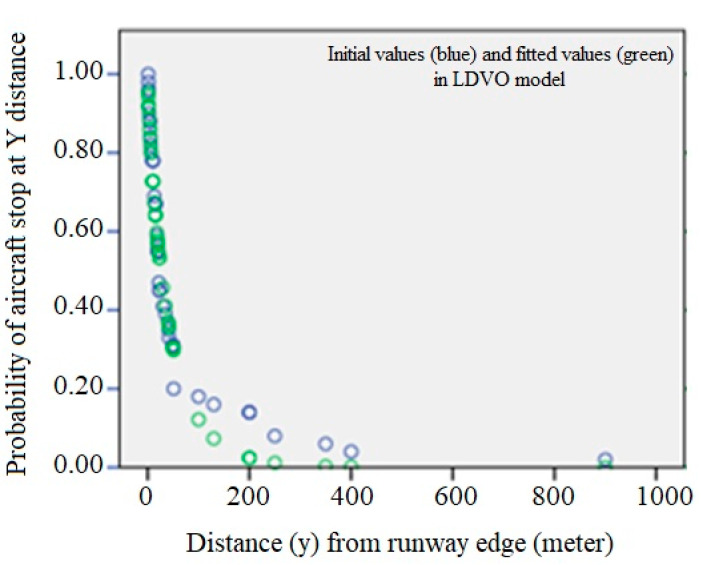
LDVO regression curve in the location accident model.

Figure 27 shows the fitted curve for the LDVO model. In this figure, the green dots, the fitted values, and the blue dots are the initial values.

**Figure 28:**
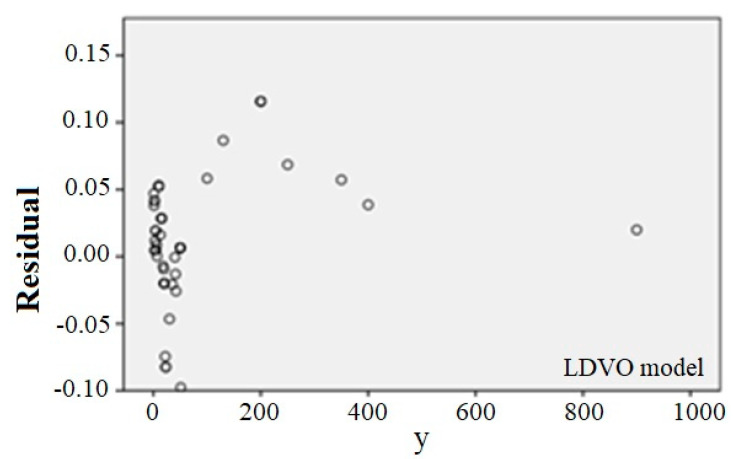
y scatter plot versus the residuals for the LDVO model.

At this step, two conditions are considered for the adequacy of the fitted model: the variance of the errors is constant and the errors are inconsistent. For this purpose, the scattering plot of the independent variable is drawn versus the residuals (Figure 28). If the model is correct, the residuals should not have a regular structure.

Figure 28 shows no significant violations of the defaults. Therefore, the selected regression model is quite appropriate.

The probability of the plane veers off from the runway edge, in the y-direction and during the landing, and locates in the range a to b from the edge of the runway is as follows:

(22) $$F_{Y}\left(y\right)=1-e^{-0.048y^{0.821}}$$

Consequently:

(23) $$P\left(a\leq y\leq b\right)=\int_{a}^{b}0.0394y^{-0.179}e^{-0.048y^{0.821}}\mathrm{dy}$$

Table 17 summarizes the models presented in this section. It should be noted that in the proposed models, the variables must be entered in the meter.

### 4.3. Accident Consequence Model

As described in Section 3.1.3, the accident consequence model for both LDOR and LDVO accidents is constructed according to the coefficients obtained in the accident location model. Based on Equations (7) and (8), Table 18 shows the accident consequence model for both LDOR and LDVO. All parameters of Table 18 are fully introduced in Section 3.1.3.

### 4.4. Practical Application of Proposed Models

Mathematical modeling of various phenomena is encountered with the success of society when it can be proved by its practical and intuitive application. In this regard, this study also examines the practical application of the proposed models. Mehrabad International Airport and Hasheminejad International Airport have been selected as case studies to review the proposed models. The general steps for using models are:

(1) Selection of the airport under review
(2) Select the runway to be examined and determine the obstacles around it
(3) Collection of NOD data related to the desired airports in at least one year, to update the accident probability model
(4) Investigate different scenarios about bopping with existing obstacles
(5) Select the flight to be studied and determine the variables required in the models
(6) Calculate the probability of a RE accident
(7) Calculate the probability of a collision with an obstacle (accident location model)
(8) Calculate the consequence probability of an accident

After inspecting both airports, the Can river at Mehrabad Airport and a Radio antenna at Hashemiehnejad Airport were selected as obstacles.

#### 4.4.1. Risk Assessment of LDOR at Mehrabad International Airport because of Can River

**Figure 29:**
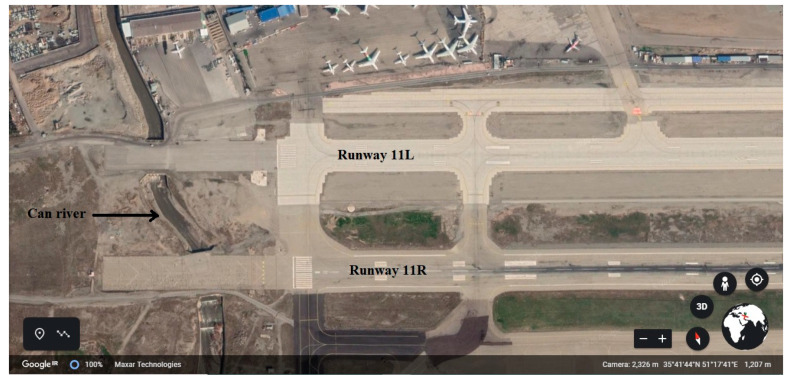
Interference of Can river with Mehrabad Airport runways.

**Figure 30:**
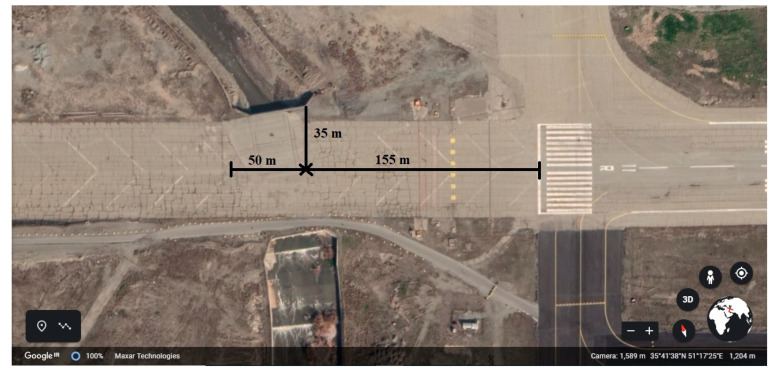
The exact location of the runway 11R and the river can interference.

The runways 11L and 11R of Mehrabad Airport overlap with the Can river which is crossed by a bridge. Figure 29 shows this area of Mehrabad Airport. In this study, the runway 11R is considered. The exact specifications of the runway 11R and the Can river are shown in Figure 30. Based on this figure, in distance between 155 to 205 m, if the aircraft deviates 35 m from the centerline of the runway to the right, it will fall into the Can river.

Then we have to choose a flight to Mehrabad airport and determine its specifications. Table 19 includes the introduction of the selected flight. After selecting the desired flight, the variables related to the probability model of accidents should be determined. Meteorological Aviation Routine Weather Report (METAR) related to Iran’s daily flights can be obtained from www.irimo.ir. Table 20 shows the variables for flight 2807.

Based on the logistics regression model presented in the Equation (4), the general form of the accident probability model is as follows:

(24) $$P\left\{\mathrm{Accident}\_\mathrm{Occurence}\right\}=\frac{1}{1+e^{-\left(b_{0}+b_{1}X_{1}+b_{2}X_{2}+b_{3}X_{3}+\ldots \right)}}=\frac{1}{1+e^{-Z}}$$

According to Table 5 and Table 6, the value of Z for LDOR accident is calculated as follows:

(25) $$\begin{array}{ll} Z=-13.065+ & 1.539\left(G\right)-0.498\left(T/C\right)-1.013\left(A/B\right)+0.935\left(D/E/F\right)\\ & -0.019\left(\mathrm{Ceiling}\;<200\mathrm{ft}\right)-0.772\left(\mathrm{Ceiling}\;200\;\mathrm{to}\;1000\;\mathrm{ft}\right)\\ & -0.345\left(\mathrm{Ceiling}\;1000\;\mathrm{to}\;2500\;\mathrm{ft}\right)+2.881\left(\mathrm{Visibility}\;<\;2\mathrm{SM}\right)\\ & +1.532\left(\mathrm{Visibility}\;2\;\mathrm{to}\;4\;\mathrm{SM}\right)+0.200\left(\mathrm{Visibility}\;4\;\mathrm{to}\;8\mathrm{SM}\right)\\ & -0.913\left(\mathrm{Xwind}\;5\;\mathrm{to}\;12\;\mathrm{kt}\right)-1.342\left(\mathrm{Xwind}\;2\;\mathrm{to}\;5\mathrm{kt}\right)\\ & -0.921\left(\mathrm{Xwind}\;\geq \;12\;\mathrm{kt}\right)+0.786\left(\mathrm{Tailwind}\;\geq \;12\mathrm{kt}\right)\\ & +0.043\left(\mathrm{Temp}\;<\;5\;C\right)-0.019\left(\mathrm{Temp}\;5\;\mathrm{to}\mathrm{15}C\right)\\ & -1.067\left(\mathrm{Temp}\;\geq \;25\;C\right)+2.007\left(\mathrm{Icing}\;\mathrm{Condition})+0.449(\mathrm{Snow}\right)\\ & -1.344(\mathrm{Thunderstorm})+0.929(\mathrm{Foreign}\;\mathrm{OD})\\ & +1.334(\mathrm{Hub}/\mathrm{Non}\;-\;\mathrm{Hub}\;\mathrm{Airport})+9.237(\mathrm{Log}\;\mathrm{Criticality}\;\mathrm{Factor}) \end{array}$$

Now, by replacing the model variables with values of 0 and 1 of the selected flight (according to Table 20), the probability of an LDOR accident at Mehrabad Airport resulting from the flight number 2807 of Meraj airlines is obtained.

(26) $$\begin{array}{ll} Z=-13.065+ & 1.539\left(0\right)-0.498\left(0\right)-1.013\left(0\right)+0.935\left(0\right)-0.019\left(0\right)-0.772\left(0\right)\\ & -0.345\left(0\right)+2.881\left(0\right)+1.532\left(0\right)+0.200\left(0\right)-0.913\left(0\right)\\ & -1.342\left(1\right)-0.921\left(0\right)+0.786\left(0\right)+0.043\left(0\right)-0.019\left(1\right)\\ & -1.067\left(0\right)+2.007\left(0\right)+0.449\left(0\right)-1.344\left(0\right)+0.929\left(0\right)\\ & +1.334\left(0\right)+9.237\left(1\right)=-5.189 \end{array}\;P\left(\mathrm{LDOR}\right)=\frac{1}{1+e^{-Z}}=\frac{1}{1+e^{-\left(-5.189\right)}}=5.547\times 10^{-3}=0.5547\%$$

As a result, the probability of overrun from runway end for the Airbus 320 of Meraj airline is 0.55%. If the aircraft overruns from the runway end, the next step is to calculate the probability of falling into the Can river. According to Table 17, the probability of falling in the Can river is equal to:

(27) $$P\left(155\leq x\leq 205\right)=\int_{155}^{255}0.0097x^{-0.196}e^{-0.012x^{0.804}}\mathrm{dx}=0.0805=8.05\%\;P\left(Y\geq 35\right)=e^{-0.932{\left(35\right)}^{0.275}}=0.0839=8.39\%$$

The probability of the aircraft overrun from the runway end and falling into the Can river is equal to:

(28) $$P\left(\mathrm{CS}\right)=P\left(\mathrm{LDOR}\right)\times P\left(155\leq x\leq 205\right)\times P\left(Y\geq 35\right)=3.7464\times 10^{-5}$$

Since the type of obstacle, in this case, is the river, so the consequences of the accident are expressed as a fall and not a collision. The fall will occur only when the aircraft deviates 35 m from the runway centerline. On the other hand, to calculate the accident with severe consequences, according to Table 18, we need two critical values: y_c_ and y_f_. Therefore, it is not possible to use the accident consequences model in this case.

#### 4.4.2. Risk Assessment of LDVO at Hasheminejad International Airport because of Radio Antenna

**Figure 31:**
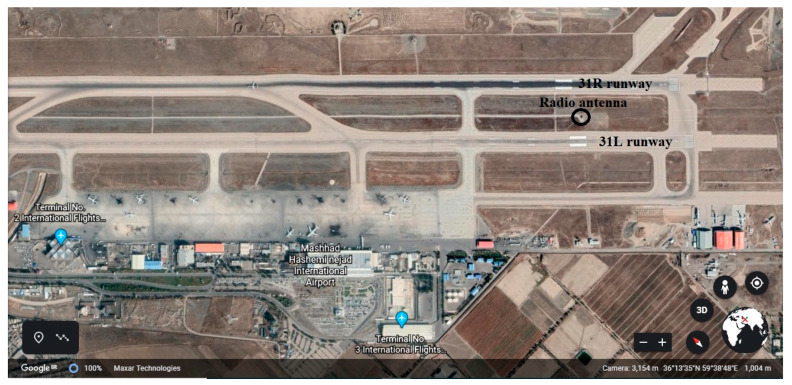
Radio antenna between runways 31L and 31R in Hasheminejad airport.

**Figure 32:**
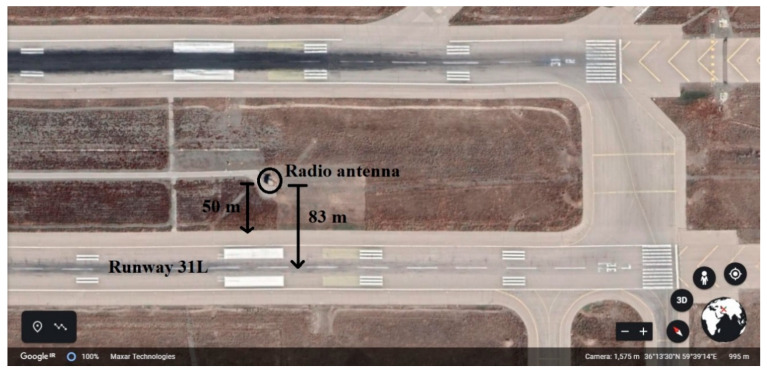
The exact location of the Radio antenna relative to runway 31L.

In this section, the possibility of colliding aircraft landing on the runways 31L and 31R with the radio antenna between these two runways is examined. Figure 31 and Figure 32 show the location of this antenna relative to the desired runway. An input flight to Hasheminejad airport has been selected and its specifications are given in Table 21. Also, Table 22 shows the variables related to the probability model of accidents on this flight.

Based on Equation (4), the general form of the accident probability model is as follows:

(29) $$P\left\{\mathrm{Accident}\_\mathrm{Occurence}\right\}=\frac{1}{1+e^{-\left(b_{0}+b_{1}X_{1}+b_{2}X_{2}+b_{3}X_{3}+\ldots \right)}}=\frac{1}{1+e^{-Z}}$$

According to Table 5 and Table 6, the value of Z for LDVO accident is calculated as follows:

(30) $$\begin{array}{ll} Z=-13.088+ & 1.682\left(G\right)-0.770\left(A/B\right)-0.252\left(D/E/F\right)+2.413\left(\mathrm{Visibility}\;<\;2\mathrm{SM}\right)\\ & +0.653\left(\mathrm{Xwind}\;5\;\mathrm{to}\;12\;\mathrm{kt}\right)-0.091\left(\mathrm{Xwind}\;2\;\mathrm{to}\;5\;\mathrm{kt}\right)\\ & +2.192\left(\mathrm{Xwind}\;\geq \;12\;\mathrm{kt}\right)+0.066\left(\mathrm{Tailwind}5\;\mathrm{to}\;12\mathrm{kt}\right)\\ & +0.980\left(\mathrm{Tailwind}\;\geq \;12\;\mathrm{kt}\right)+0.558\left(\mathrm{Temp}\;<\;5\;C\right)\\ & -0.453\left(\mathrm{Temp}\;5\;\mathrm{to}\;15C\right)+0.291\left(\mathrm{Temp}\;\geq \;25\;C\right)\\ & +2.670\left(\mathrm{Icing}\;\mathrm{Condition}\right)-\;0.126\left(\mathrm{Gusts})\;+\;0.548(\mathrm{Snow}\right)\\ & -0.103\left(\mathrm{Frozen}\;\mathrm{Precipitation}\right)-0.036\left(\mathrm{Gusts})\;+\;1.74(\mathrm{Fog}\right)\\ & -2.517\left(\mathrm{Turboprop}\right)\;-\;0.334\left(\mathrm{Foreign}\;\mathrm{OD}\right)\\ & +4.318\left(\mathrm{Log}\;\mathrm{Criticality}\;\mathrm{Factor}\right)\;-\;1.360\left(\mathrm{Night}\;\mathrm{Condition}\right) \end{array}$$

Now, by replacing the model variables with values of 0 and 1 of the selected flight (according to Table 22), the probability of an LDVO accident at Mehrabad Airport resulting from the flight number 6254 of Taban airlines is obtained.

(31) $$\begin{array}{ll} Z=-13.088+ & 1.682\left(0\right)-0.770\left(0\right)-0.252\left(0\right)+2.143\left(0\right)+0.653\left(0\right)-0.091\left(0\right)\\ & +2.192\left(0\right)+0.066\left(0\right)+0.980\left(0\right)+0.558\left(1\right)-0.453\left(0\right)\\ & +0.291\left(0\right)+2.670\left(0\right)-0.126\left(0\right)+0.548(0)-0.103\left(0\right)\\ & -0.036\left(0\right)+1.74\left(0\right)-2.517\left(0\right)-0.334\left(0\right)+4.318\left(1\right)-1.360\left(1\right)\\ & =-9.572 \end{array}$$

(32) $$P\left(\mathrm{LDVO}\right)=\frac{1}{1+e^{-Z}}=\frac{1}{1+e^{-\left(-9.572\right)}}=6.965\times 10^{-5}=0.006965\%$$

As a result, the probability of deviation from the runway edge for flight 6254 of Taban airline is 0.01 percent. According to Table 17, if aircraft deviates from the runway, the probability of collision it with the radio antenna is:

(33) $$P\left(Y\geq 50\right)=e^{-0.048{\left(50\right)}^{0.821}}=0.3038=30.38\%$$

Therefore:

(34) $$P\left(\mathrm{CS}\right)=P\left(\mathrm{LDVO}\right)\times P\left(Y\geq 50\right)=2.115\times 10^{-3}$$

Therefore, it can be said that the probability of Taban airlines flight 6254 deviates from the edge of the runway and collides with the radio antenna of Hasheminejad airport is equal to 0.002115.

**Figure 33:**
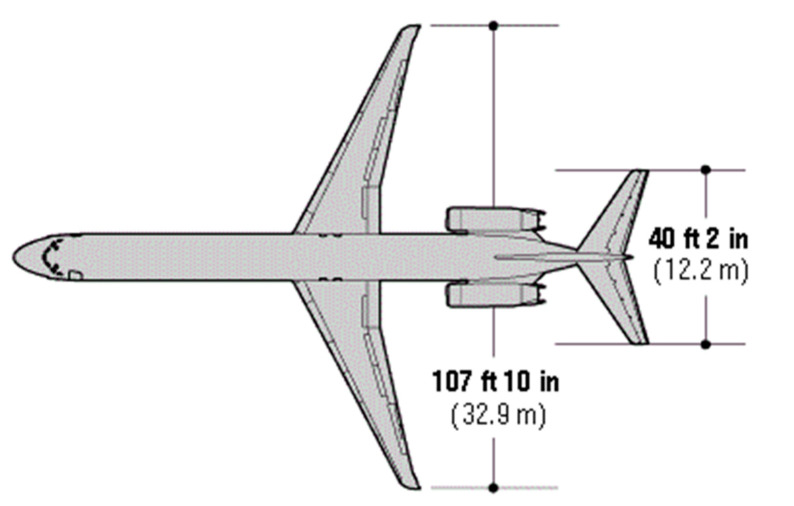
Dimensions of aircraft MD-88.

About the probability of an accident with severe consequences, critical transverse distances (y_c_ and y_f_) must first be obtained. These distances are determined with the help of aircraft dimensions (See Figure 33).

**Figure 34:**
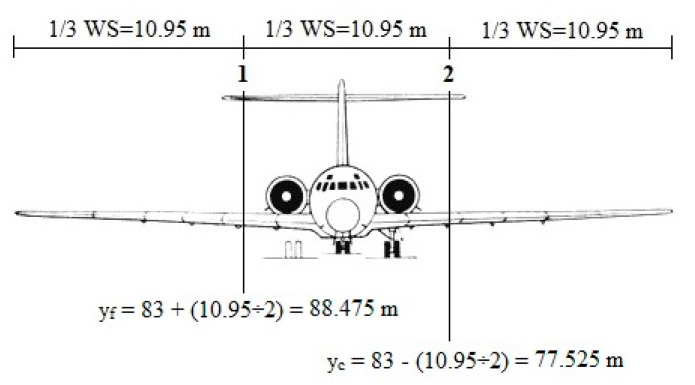
Determination of critical aircraft location for consequences model. Note: Distance from radio antenna to runway axis is 83 m (See Figure 32). Also, when the obstacle is at positions 1 and 2, distance from nose wheel to runway axis names y_f_ and y_c_, respectively.

According to Figure 33, the wingspan of the aircraft MD-88 is 32.9 m. According to Figure 12, the consequences of colling with an obstacle is severe when the obstacle is located in the orange area (middle 1/3 wingspan). Figure 34 shows the critical positions of the obstacle over the MD-88 aircraft.

As shown in Figure 34, y_c_ is 77.525 m. With a similar argument, y_f_ is equivalent to 88.475 m. Based on the equations in Table 19:

(35) $$P_{\mathrm{sc}}=\frac{e^{-0.048y_{c}^{0.821}}-e^{-0.048y_{f}^{0.821}}}{2}=\frac{e^{-0.048{\left(77.525\right)}^{0.821}}-e^{-0.048{\left(88.475\right)}^{0.821}}}{2}=0.0161$$

If the aircraft collides with the radio antenna, 1.61% of accidents will likely occur with severe consequences. As a result, the probability of an accident with severe consequences due to the departure of aircraft No. 6254 from the side of the runway 31L of Hasheminejad airport and collision with the radio antenna is equal to:

(36) $$P\left(\mathrm{LDVO}\;\mathrm{Severe}\right)=P\left(\mathrm{LDVO}\right)\times P\left(Y\geq 50\right)\times P_{\mathrm{sc}}=3.40515\times 10^{-5}$$

The evaluation of Mehrabad and Hasheminejad airports verified that presented models can be used well in Iran. After setting the risk threshold by the airport officials, it is possible to make the necessary decisions and budget for the next steps. For example, the acceptable level of risk at Mehrabad Airport may be such that a decision is made to cover the Can river. But the risk threshold at Hasheminejad Airport may be such that there is no need to move the radio antenna. Therefore, the models presented in this paper can be a good criterion for evaluating the obstacles around the runway to prevent unprofessional, unsafe, and uneconomical decisions. In general, these accident assessment models will improve the performance of the air transportation system, both in terms of safety and economics. In other words, these models help engineers make the most optimal decisions and always be in the position that has the safest and most economical conditions possible. Besides, the models presented in this paper help to eliminate the permanent lack of a proper risk assessment system for runways at Iranian airports.

## 5. Conclusions

In today’s world, the development and advancement of the air transportation industry are evident. Airports are one of the most important and vital centers. Runways are also one of the main physical parts of airports. Accidents on runways cause them to be blocked. Blocking the runway also significantly reduces airport capacity (At airports with one runway, capacity drops to zero). Also, these accidents may danger human lives and environmental elements. Therefore designing safer runways is of utmost importance. The primary goal of this study is to achieve a risk assessment model of RSA. A review of studies and research in this field showed that only the United States has been able to achieve a comprehensive and complete model for assessing the risk of accidents and incidents resulting from the departure of aircraft from runways. Therefore, an attempt was made to calibrate the model for Iranian airports.

Focusing on RE accidents and carefully examining predictive models of the probability of occurrence, location, and severity of consequences when smashing with obstacles have been the mainstays of this study. Therefore, after reviewing much research conducted in this field, an attempt was made to compile a well-written and complete process to collect data from modeling RE accidents in Iran. In this way, the lack of systematic documentation and the lack of access to the codes of NOD flights led to the inability to build a model of the probability of accidents. Subsequently, the accuracy of the constructed models was confirmed by statistical tests. In the end, Mehrabad International Airport in Tehran and Hasheminejad International Airport in Mashhad were examined by the models. Compared to the comprehensive and famous model provided in the United States, the probability model in this paper is the same because there was no suitable database for NOD flights in Iran. In location and consequences models, the general form of the models was chosen similar to the American model, but the model coefficients presented in this paper are different from the American model coefficients. In fact, the model presented by the authors has been calibrated for Iran and allows the runway risk assessment to be done more accurately. Now, using the presented models, we can assess the risk of obstacles around the runways of airports and decide about them based on the level of risk and the requirements of the conditions. New airports can also be located in such a way that we have the least risk in the event of aircraft accidents. This decision is very important in times of crisis because it can have a significant impact on the continuity of airport services. In the long-term, these models will help increase public health and protect the environment.

The most important limitation of this study was the lack of access to NOD flight data. Therefore, for future studies, the authors suggest designing and setting up an accurate system for recording all the necessary information. In this case, the model of the probability of accidents can be calibrated for Iran. The authors also believe that adding new metrics such as obstacle type and dimensions, obstacles density, etc. in future studies can improve models and make them more comprehensive. Another idea that the authors have in mind for future studies is to develop their modeling so that other types of accidents, including LDUS, TOOR, and TOVO, can be evaluated.

## Figures and Tables

**Table 1:** Veer-off modeling.

Model	Model Application
$P=1.37\times 10^{-7}\times e^{-0.0219x}$	valid for landings on instrument runways
$P=9.72\times 10^{-7}\times e^{-0.0219x}$	valid for landings on non-instrument runways
$P=6.96\times 10^{-8}\times e^{-0.0143x}$	valid for take-offs from instrument runways
$P=6.82\times 10^{-9}\times e^{-0.0143x}$	valid for take-offs non-instrument runways

**Table 2:** The proposed methodology for risk assessment of runway landing overruns.

Model	Formula	Description
A. Modeling the probability of an overrun	$P\left(\mathrm{landing}\;\mathrm{overrun}\right)=\frac{1}{1+e^{-\left(m+\mathrm{nD}\right)}}$ P(no landing overrun)=1−P(landing overrun)	P is the probability of an overrun, D is either the percent of excess distance available or percent of maximum allowable weight, m and n are constants to be determined.
B. Modeling the wreckage location	L = 15 + 1.05 E	L is the normalized wreckage location relative to the end of the normalized required landing distance expressed as a percentage of the required landing distance, E is the excess distance between the end of the required landing distance and the runway end expressed as a percentage of the required landing distance.
C. Modeling the damage consequences of overruns *	$P\left(\mathrm{sd}\right)=\frac{1}{e^{-\left(g+\mathrm{hB}\right)}}$ P(nm)=1−P(sd)	P(sd) is the probability of the aircraft suffering substantial damage or being destroyed, P(nm) is the probability of the aircraft suffering no damage or minor damage, B signifies if the aircraft strikes an obstacle beyond the runway end (yes = 1, no = 0), g and h are parameters.
	$P\left(d\right)=\frac{1}{e^{-\left(i+{\mathrm{jB}}_{2}+\mathrm{kS}\right)}}$ P(s)=1−P(d)	P(d) is the probability of the aircraft being destroyed, P(s) is the probability of the aircraft being seriously damaged, B_2_ signifies if the aircraft struck a second obstacle beyond the runway end (yes = 1, no = 0), S is the runway exit speed in meters per second, and i, j, and k are parameters.

* The most reliable predictors of whether the aircraft will suffer substantial damage or be destroyed are whether the aircraft has struck a second obstacle beyond the runway end, and the runway exit speed, so the second stage model is similar but with two variables.

**Table 3:** Coefficients of accident location model.

Type of Accident	Type of Data	Model	R^2^
LDOR	X	$P\left\{d>x\right\}=e^{-0.00321x^{0.98494}}$	0.998
	Y	$P\left\{d>y\right\}=e^{-0.20983x^{0.48620}}$	0.939
LDUS	X	$P\left\{d>x\right\}=e^{-0.00148x^{0.75150}}$	0.987
	Y	$P\left\{d>y\right\}=e^{-0.02159x^{0.77390}}$	0.986
LDVO	Y	$P\left\{d>y\right\}=e^{-0.02568x^{0.80395}}$	0.915
TOOR	X	$P\left\{d>x\right\}=e^{-0.00109x^{1.06764}}$	0.992
	Y	$P\left\{d>y\right\}=e^{-0.04282x^{0.65957}}$	0.987
TOVO	Y	$P\left\{d>y\right\}=e^{-0.01639x^{0.86346}}$	0.942

**Table 4:** Advanced location model.

Type of Accident	Type of Data	Model	R^2^
LDOR	X	$P\left\{d>x\right\}=e^{-0.00321x^{0.984932}}$	0.998
	Y	$P\left\{d>y\right\}=e^{-0.20983x^{0.48621}}$	0.942
LDUS	X	$P\left\{d>x\right\}=e^{-0.01481x^{0.749897}}$	0.986
	Y	$P\left\{d>y\right\}=e^{-0.02159x^{0.78789}}$	0.989
LDVO	Y	$P\left\{d>y\right\}=e^{-0.2568x^{0.803984}}$	0.994
TOOR	X	$P\left\{d>x\right\}=e^{-0.00109x^{1.06754}}$	0.989
	Y	$P\left\{d>y\right\}=e^{-0.04282x^{0.661398}}$	0.991
TOVO	Y	$P\left\{d>y\right\}=e^{-0.01639x^{0.874332}}$	0.943

**Table 5:** Independent variables (X_i_) in the accident probability model.

Independent Variables (X_i_)	Levels of Variable
User Class	Commercial (C) (Base level *)
	Cargo (F)
	General Aviation (GA)
	Taxi/Commuter (T/C)
Equipment Class based on Maximum Takeoff Weight (MTOW)	Large Jet (B737, A320, etc.) (C) (Base level)
	Heavy Jet (B777, A340, etc.) (AB)
	Large Commuter (Regional Jets, ERJ-190, CRJ-900, ATR42, etc.) (D)
	Medium Aircraft (Biz Jets, Embraer120, Learjet35, etc.) (E)
	Small Aircraft (Beech-90, Cessna Caravan, etc.) (F)
Engine Type	Jet (Base level)
	Turboprop
Foreign Origin/Destination (Foreign O/D)	Domestic
	Foreign
Ceiling Height	More than 2500 ft (Base level)
	Less than 200 ft
	Between 200 ft to 1000 ft
	Between 1000 ft to 2500 ft
Visibility	Visibility is more than 8 miles (Base level)
	Visibility is less than 2 miles
	Visibility is between 2 miles and 4 miles
	Visibility is between 4 miles and 8 miles
Crosswind (Xwind)	Less than 2 knots (Base level)
	Between 2 knit and 5 knots
	Between 5 knit and 12 knots
	More than 12 knots
Tailwind	Less than 5 knots (Base level)
	Between 5 knit and 12 knots
	More than 12 knots
Air Temperature	Between 15 °C to 25 °C (Base level)
	Less than 5 °C
	Between 5 °C to 15 °C
	More than 25 °C
Gust	Non-existence (Base level)
	Existence
Thunderstorm	Non-existence (Base level)
	Existence
Rain	Non-existence (Base level)
	Existence
Snow	Non-existence (Base level)
	Existence
Fog	Non-existence (Base level)
	Existence
Icing Condition	Non-existence (Base level)
	Existence
Frozen Precipitation	Non-existence (Base level)
	Existence
Hub/Non-Hub Airport	Hub Airport (Base level)
	Non-Hub Airport
Log Criticality Factor	CF < 0 (Base level)
	CF > 0
Night Condition	It is not night (Base level)
	It is night

* The base levels in this table do not affect the model and are part of the ideal conditions.

**Table 6:** Values of b_i_ coefficients for the accident probability model.

Variable	LDOR	LDVO	LDUS	TOOR	TOVO
Adjusted Constant	−13.065	−13.088	−15.378	−14.293	−15.612
User Class F	-	-	1.693	1.266	-
User Class G	1.539	1.682	1.288	-	2.094
User Class T/C	−0.498	-	0.017	-	-
Aircraft Class A/B	−1.013	−0.770	−0.778	−1.150	−0.852
Aircraft Class D/E/F	0.935	−0.252	0.138	−2.108	−0.091
Ceiling less than 200 ft	−0.019	-	0.07	0.792	-
Ceiling 200 to 1000 ft	−0.772	-	−1.144	−0.114	-
Ceiling 1000 to 2500 ft	−0.345	-	−0.721	-	-
Visibility less than 2 SM	2.881	2.143	3.096	1.364	2.042
Visibility from 2 to 4 SM	1.532	-	1.824	−0.334	0.808
Visibility from 4 to 8 SM	0.2	-	0.416	0.652	−1.500
Xwind from 5 to 12 kt	−0.913	0.653	−0.295	−0.695	0.102
Xwind from 2 to 5 kt	−1.342	−0.091	−0.698	−1.045	-
Xwind more than 12 kt	−0.921	2.192	−1.166	0.219	0.706
Tailwind from 5 to 12 kt	-	0.066	-	-	-
Tailwind more than 12 kt	0.786	0.98	-	-	-
Temp less than 5 C	0.043	0.558	0.197	0.269	0.988
Temp from 5 to 15 C	−0.019	−0.453	−0.710	−0.544	−0.420
Temp more than 25 C	−1.067	0.291	−0.463	0.315	−0.921
Icing Condition	2.007	2.67	2.703	3.324	-
Rain	-	−0.126	0.991	0.355	−1.541
Snow	0.449	0.548	−0.250	0.721	0.963
Frozen Precipitation	-	−0.103	-	-	-
Gusts	-	−0.036	0.041	0.006	-
Fog	-	1.74	-	-	-
Thunderstorm	−1.344	-	-	-	-
Turboprop	-	−2.517	-	0.56	1.522
Foreign OD	0.929	−0.334	1.354	-	−0.236
Hub/Non−Hub Airport	1.334	-	-	-	−0.692
Log Criticality Factor	9.237	4.318	1.629	-	1.707
Night Condition	-	−1.360	-	-	-

**Table 7:** Air accident rates based on IATA zoning (per one million operations).

Zone	Air accident Rates in 2013	Air Accident Rates in 2014	Average Air Accident Rates (2009–2013)
AFI	11.18	7.12	12.45
ASPAC	2.57	2.90	2.76
CIS	2.19	3.14	5.92
EUR	1.35	2.75	2.03
LATAM	2.73	1.98	3.36
MENA	3.47	3.05	5.43
NAM	1.00	1.55	1.38
NASIA	0.95	0.53	0.82

**Table 8:** List of selected countries and the number of data usable from each country.

MENA	Number of Data	CIS	Number of Data	LATAM	Number of Data	ASPAC	Number of Data
Iran	13	Russia	65	Brazil	21	Indonesia	47
Afghanistan	4	Ukraine	8	Colombia	11	India	13
Sudan	3	Kazakhstan	5	Argentina	8	Japan	8
Morocco	2	Tajikistan	2	Bolivia	6	Malaysia	7
Tunisia	2	Uzbekistan	2	Ecuador	6	Philippines	6
UAE.	2	Armenia	1	Mexico	6	Australia	5
Yemen	2	Georgia	1	Venezuela	3	Bangladesh	4
Algeria	1	Kyrgyzstan	1	Guatemala	2	Nepal	4
Iraq	1			Honduras	2	Pakistan	4
Saudi Arabia	1			Nicaragua	2	Singapore	4
				Peru	2	New Guinea	2
				The Bahamas	1	South Korea	2
				Belize	1	Thailand	2
				Chile	1	Vietnam	2
				Cuba	1	Brunei	1
				Dominican Republic	1	Laos	1
				Guyana	1	Maldives	1
				Haiti	1	New Zealand	1
				Jamaica	1	Sri Lanka	1
				Panama	1		
SUM	31	SUM	85	SUM	78	SUM	115

**Table 9:** Physical characteristics of Mehrabad International Airport runways.

Runway	Dimensions (m)	Pavement Type	Stopway	Strip	RESA
11L	3989 × 45	Asphalt	122 × 45	-	226 × 150
29R	3989 × 45	Asphalt	194 × 45	-	-
11R	4030 × 60	Asphalt	87 × 45	-	-
29L	4030 × 60	Asphalt	-	-	-

Note: RESA is Runway End Safety Area.

**Table 10:** Physical characteristics of Hasheminejad International Airport runways.

Runway	Dimensions (m)	Pavement Type	Stopway	Strip	RESA
13L	3810 × 45	Asphalt	302 × 45	-	-
31R	3810 × 45	Asphalt	303 × 45	-	-
13R	3920 × 45	Asphalt	300 × 45	-	-
31L	3920 × 45	Asphalt	296 × 45	-	-

Note: RESA is Runway End Safety Area.

**Table 11:** Estimation of unknown parameters of the LDOR model in the X-direction.

Unknown Parameter	Estimated Value	Standard Error of the Mean (SE)	95% Confidence Interval	
			Lower Bound	Upper Bound
b_0_	0.012	0.001	0.011	0.014
b_1_	0.804	0.011	0.781	0.827

**Table 12:** Analysis of Variance (ANOVA) for the LDOR model in the X-direction.

Source of Variations	Sum of Squares	Degree of Freedom (df)	Mean Squares
Regression	44.340	2	22.170
Residual	0.095	126	0.001
Uncorrected Total	44.434	128	-
Corrected Total	10.876	127	-

**Table 13:** Estimation of unknown parameters of the LDOR model in the Y-direction.

Unknown Parameter	Estimated Value	Standard Error of the Mean (SE)	95% Confidence Interval	
			Lower Bound	Upper Bound
b_0_	0.932	0.018	0.897	0.966
b_1_	0.275	0.005	0.264	0.286

**Table 14:** Analysis of Variance (ANOVA) for the LDOR model in the Y-direction.

Source of Variations	Sum of Squares	Degree of Freedom (df)	Mean Squares
Regression	123.142	2	61.571
Residual	0.001	141	0.000
Uncorrected Total	123.143	143	-
Corrected Total	14.767	142	-

**Table 15:** Estimation of unknown parameters of the LDVO model.

Unknown Parameter	Estimated Value	Standard Error of the Mean (SE)	95% Confidence Interval	
			Lower Bound	Upper Bound
b_0_	0.048	0.006	0.036	0.061
b_1_	0.821	0.039	0.743	0.898

**Table 16:** Analysis of Variance (ANOVA) for the LDVO model.

Source of Variations	Sum of Squares	Degree of Freedom (df)	Mean Squares
Regression	17.461	2	8.731
Residual	0.118	47	0.003
Uncorrected Total	17.579	49	-
Corrected Total	4.078	48	-

**Table 17:** Summarize of accident location model.

Accident Type	Point Model	Interval Model	R^2^
LDOR (X)	$P\left(X\geq x\right)=e^{-0.012x^{0.804}}$	$P\left(a\leq x\leq b\right)=\int_{a}^{b}0.0097x^{-0.196}e^{-0.012x^{0.804}}\mathrm{dx}$	0.991
LDOR (Y)	$P\left(Y\geq y\right)=e^{-0.932y^{0.275}}$	$P\left(a\leq y\leq b\right)=\int_{a}^{b}0.2563y^{-0.725}e^{-0.932y^{0.275}}\mathrm{dy}$	1.00
LDVO	$P\left(Y\geq y\right)=e^{-0.048y^{0.821}}$	$P\left(a\leq y\leq b\right)=\int_{a}^{b}0.0394y^{-0.179}e^{-0.048y^{0.821}}\mathrm{dy}$	0.971

**Table 18:** Accident consequence model.

Accident Type	Interval Model
LDOR	$P_{\mathrm{sc}}=\sum^{\;}\frac{\left(e^{-0.932y_{c}^{0.275}}-e^{-0.932y_{f}^{0.275}}\right)}{2}\times e^{-0.012{\left(x_{i}+\Delta_{i}\right)}^{0.804}}$
LDVO	$P_{\mathrm{sc}}=\frac{e^{-0.048y_{c}^{0.821}}-e^{-0.048y_{f}^{0.821}}}{2}$

**Table 19:** Selected flight specifications at Mehrabad Airport.

Origin	Mashhad
Destination	Tehran
Date	02.12.2016
Landing time in the destination	18:58
Flight number	2807
Airline	Meraj
Aircraft type	Airbus 320

**Table 20:** Variables related to the probability model of RE accidents for flight 2807.

Variable	Value or Type	Description
User Class	C	Commercial
Maximum Takeoff Weight (MTOW)	C	Large Jet (B737, A320, etc.)
Equipment Class	Jet	-
Foreign Origin/Destination	Domestic	-
Ceiling Height	+2500	More than 2500 ft
Visibility	+8	More than 8 miles
Crosswind	Between 2 and 5	3 knots
Tailwind	Less than 5	3 knots
Air Temperature	Between 5 and 15	6 ºC
Gust	Do not exist	-
Thunderstorm	Do not exist	-
Rain	Do not exist	-
Snow	Do not exist	-
Fog	Do not exist	-
Icing Condition	Do not exist	-
Frozen Precipitation	Do not exist	-
Hub/Non-Hub Airport	Hub	-
Log Criticality Factor	CF > 0	Needed length is 6049 ft
Night Condition	It is night	-

**Table 21:** Selected flight specifications at Hasheminejad Airport.

Origin	Tehran
Destination	Mashhad
Date	02.13.2016
Landing time in the destination	20:03
Flight number	6254
Airline	Taban
Aircraft type	McDonnell Douglas MD-88

**Table 22:** Variables related to the probability model of RE accidents for flight 6254.

Variable	Value or Type	Description
User Class	C	Commercial
Maximum Takeoff Weight (MTOW)	C	Large Jet (B737, A320, etc.)
Equipment Class	Jet	-
Foreign Origin/Destination	Domestic	-
Ceiling Height	+2500	More than 2500 ft
Visibility	Between 5 and 15	3.72 miles
Crosswind	Less than 2	1 knot
Tailwind	Less than 5	4 knots
Air Temperature	Less than 5	−2 °C
Gust	Do not exist	-
Thunderstorm	Do not exist	-
Rain	Do not exist	-
Snow	Do not exist	-
Fog	Do not exist	-
Icing Condition	Do not exist	-
Frozen Precipitation	Do not exist	-
Hub/Non-Hub Airport	Hub	-
Log Criticality Factor	CF > 0	Needed length is 5813 ft
Night Condition	It is night	-
